# 1,1-Diphenyl-2-picrylhydrazyl radical and superoxide anion scavenging activity of *Rhizophora mangle* (L.) bark

**DOI:** 10.4103/0974-8490.72323

**Published:** 2010

**Authors:** Janet Calero Sánchez, Roberto Faure García, Ma. Teresa Mitjavila Cors

**Affiliations:** *Pharmacology and Toxicology Group, National Center of Animal and Plant Health (CENSA), Carretera de Tapaste y Autopista Nacional, Aptdo. #10, San José de Las Lajas, La Habana, Cuba*; 1 *Department of Physiology, Faculty of Biology, University of Barcelona, Barcelona, Spain*

**Keywords:** Antioxidant activity, DPPH, polyphenolic compounds, *Rhizophora mangle* (L.), superoxide anions

## Abstract

**Background:**

*Rhizophora mangle* (L.) produce a variety of substances that possesses pharmacological actions. Although it shown antioxidant properties in some assays, there is no available information about its effect on some free radical species. So the objective of the present research is to evaluate the DPPH radical and superoxide anion scavenging properties of *R. mangle* extract and its polyphenol fraction.

**Methods:**

*Rhizophora mangle* (L.) bark aqueous extract and its major constituent, polyphenols fraction, were investigated for their antioxidant activities employing 2 *in vitro* assay systems: 1,1-diphenyl-2-picrylhydrazyl (DPPH) and superoxide anion radicals scavenging.

**Results:**

IC_50_ for DPPH radical-scavenging activity was 6.7 *µ*g tannins/mL for extract and 7.6 *µ*g tannins/mL for polyphenolic fraction. The extract showed better activity than its fraction (*P* < 0.05) in the DPPH radicals reducing power. Polyphenolic fraction exhibited better superoxide anion scavenging ability (IC_50_ = 21.6 *µ*g tannins/mL) than the extract (IC_50_ = 31.9 *µ*g tannins/mL). Antioxidant activities of both samples increased with the rise of tannins concentration. The comparison of regression lines showed significant differences (*P* < 0.05) between extract and its polyphenolic fraction in both assays, indicating that extract was more effective in DPPH radical scavenging than its fraction at tannin concentrations below the crossing point of both lines, while that fraction was more effective than extract inhibiting the superoxide anions generation.

**Conclusions:**

*R. mangle* aqueous extract showed a potent antioxidant activity, achieved by the scavenging ability observed against DPPH radicals and superoxide anions. Regarding its polyphenolic composition, the antioxidant effects observed in this study are due, most probably, to the presence of polyphenolic compounds.

## INTRODUCTION

The term antioxidant refers to the activity of numerous vitamins, minerals, and other phytochemicals to protect against the damage caused by reactive oxygen species (ROS). By their ability to react and damage many structures in the body, ROS are involved in various related physiologic processes and diseases, such as aging, cancer, and atherosclerosis.[1]

Several studies have demonstrated that plants produce potent antioxidants and represent an important source of natural antioxidants. Among these, *Rhizophora mangle* (L.), is a characteristic tree of the lower and swampy zones of Cuba. Several medicinal properties are attributed to this tree in traditional medicine. It has been used to treat diseases of the throat and pulmonary tuberculosis,[2] leprosy, asthma, contaminated fish poisoning, peptic ulcer, digestive disorders, skin infections, and sexually transmitted diseases.[3] Also it was reported to have antifungal activity.[4]

The chemical composition of the aqueous extract from *R. mangle* bark revealed the presence of polyphenols (54%), represented by polymeric tannins (80%) and hydrolysable tannins (20%); special emphasis has been given to the presence of epicatechin, catechin, chlorogenic, gallic, and elagic acids, as well as, galotannins, elagitannins, and condensed tannins. Moreover, this extract also contains nontannin structures of free and bound carbohydrates (xylose, ramnose, fucose, glucose, arabinose, mannose, and galactose), saturated and unsaturated long-chain fatty acids from C 12:0 to C 24:0, essential oils, and phytosterols.[5]

In the last decade, aqueous extract from *R. mangle* bark, has shown several pharmacologic properties, which include the following: prevention of bovine mastitis,[6] efficacy in healing wounds,[7,8] antimicrobial properties,[9,10] effective in the treatment of uterine infections[11] and gastroduodenal ulcers,[12] and it also showed anti-inflammatory properties.[13]

Although some antioxidant properties of this plant have been demonstrated previously, such as inhibition of lipid peroxidation in rat brain, reduction in the risk of free radical-induced hemolysis,[14] hydroxyl radical-scavenging activity, iron chelating property[15]; and in an aseptic open wound healing model in rats, increased antioxidant indicators: superoxide dismutase, catalase, reduced glutathione; and decreased prooxidant biomarkers: malondialdehyde, during wound reparation process,[16] there is no available information about its effect on other free radical species, such as: 1,1-diphenyl-2-picrylhydrazyl (DPPH) and superoxide anion (O_2_^&rad;–^), an aspect that is important to know to complete the spectrum of action of *R. mangle* extract as a free radical scavenger and to elucidate its mechanism of action in this direction. So, the objective of the present research is to evaluate the DPPH radical and superoxide anion scavenging properties of *R. mangle* extract and its polyphenol fraction.

## MATERIALS AND METHODS

### Plant material

*R. mangle* (L.) was collected from the region of Caimito Beach (Havana, Cuba) in June 2007. The botanical identification was performed in the Herbarium of National Botanical Garden (Havana City, Cuba). A voucher specimen is kept in the herbarium of the Pharmacology Department, CENSA.

### Preparation of plant extract

Fresh barks were washed and air-dried at room temperature (25°C). The extract of *R. mangle* was obtained from the bark aqueous extraction at 95°C for 30 min, in a reactor of 250-L capacity, in 1:7 (w/v) ratio. The concentration was adjusted to 24 mg/mL of dry material, with tannin content higher than 12 mg/mL. Quality of each batch was strictly controlled by the Quality Assurance Department of CENSA.

### Preparation of polyphenolic fraction

The polyphenolic fraction was obtained from *R. mangle* aqueous extract at a concentration of 24 mg /mL by precipitation with NaCl, in 1:2.8 (w/v) ratio.[17] NaCl was added slowly to the extract, under conditions of agitation and heating at 70°C for 10 min. The supernatant was discarded and the samples were centrifuged at 3500 rpm for 15 min at room temperature. The precipitate was dissolved in water and it was subjected to 3 extractions with *n*-butanol (1:2, v/v) to remove contamination of salts. The butanol phase was collected and it was evaporated in a rotary evaporator to dryness to eliminate the organic solvent. The obtained material was lyophilized and stored at –20°C.

### Determination of tannins content

Tannins content in the *R. mangle* aqueous extract and its polyphenols fraction was estimated as previously described.[18] Briefly, this colorimetric method measures the amount of precipitated tannins by a standard protein, bovine serum albumin (BSA). The reaction mixture contained *R. mangle* extract, its polyphenols fraction or tannic acid as standard (1 mL), and BSA (1 mg/mL), which were mixed in vortex and put to rest for 15 min at room temperature. Then, it was centrifuged at 5500 rpm for 15 min and then the supernatant was poured off. The pellet was dissolved in 15 mL of lauryl sodium sulfate (1%) and 1 mL of it was added to 3 mL of a solution that contained lauryl sodium sulfate (1%) and triethanolamine (7%), and 1 mL FeCl_3_ (0.01 M in HCl 0.1 N). Immediately the mixture was shaken in the vortex. After 15 min, the absorbance was read at 510 nm and the tannin concentration in the *R. mangle* extract or its polyphenol fraction was calculated using a patron curve with tannic acid as the standard.

### DPPH radical-scavenging activity

The production of DPPH radicals was evaluated as previously described.[19] Briefly, reaction mixtures contained 100 µM DPPH in ethanol solution with or without each sample in a final volume of 3.0 mL. Various concentrations of *R. mangle* extract (4.2, 8.5, 17.0 µg/mL of tannins) and its fraction (2.3, 4.6, 9.2, 18.3 µg/mL of tannins) were evaluated. Butylated hydroxytoluene (BHT) at 22.0 µg/mL and phloroglucinol at 37.8 µg/mL were used as standard antioxidants. After incubation at 37°C for 10 min, the absorbance was measured at 517 nm. The DPPH radical-scavenging activity of *R. mangle* extract and its polyphenolic fraction was determined by decreasing the absorbance values. The results were expressed as inhibition percentage of DPPH radical formation.

### Superoxide anions-scavenging activity

Superoxide radicals generated by the xanthine–xanthine oxidase system was determined spectrophotometrically by monitoring the product of nitroblue tetrazolium (NBT).[20] Various concentrations of the extract (4.2, 8.5, 17.0, 34.0 µg/mL of tannins) and its fraction (2.3, 4.6, 9.2, 18.3 µg/mL of tannins) were added to the reaction mixture containing: 3.0 mM xanthine, 3.0 mM ethylenediaminetetraacetic acid, 0.75 mM NBT, 50 mU/mL xanthine oxidase, and 50 mM carbonate buffer (pH 10.2), making up a final volume of 2.4 mL. After incubation of the mixture at 25°C for 10 min, the absorbance was read at 560 nm and compared with the control samples, containing the enzyme, xanthine oxidase, but the samples were not included. The percentage of superoxide anions scavenging was calculated from the optical density of the treated and control samples.

### Statistical analysis

Experimental results are expressed as means ± standard error of mean. All the measurements were replicated 3 times. The data were analyzed by a linear regression analysis to obtain the regression curves relating the tannins concentration with the free radical-scavenging effect of the *R. mangle* extract and its polyphenolic fraction, and subsequently the comparison of them, using the statistical package, STATGRAPHICS version 4.1 for Windows (USA), considering *P* < 0.05 as statistically significant.

## RESULTS

**Figure 1:**
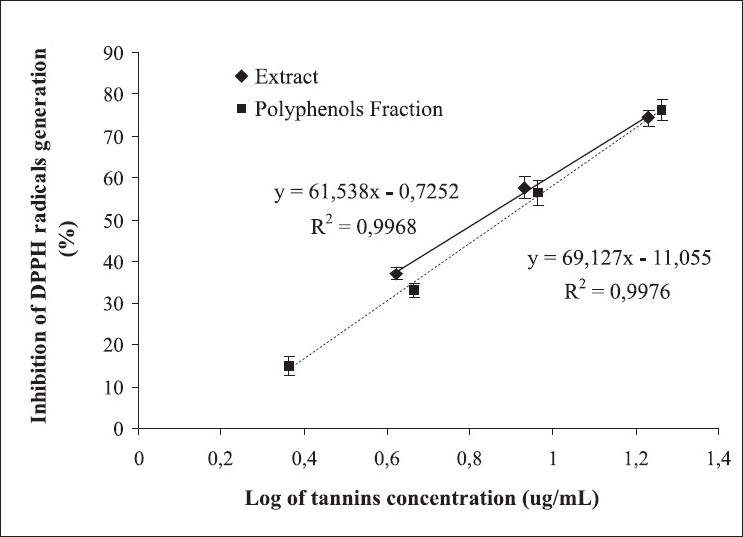
Effect of tannins concentration in the *Rhizophora mangle* L. aqueous extract and its polyphenolic fraction on DPPH radical generation. The butylated hydroxytoluene (concentration: 22.0 µg/mL, percentage of inhibition: 29.7) and phloroglucinol (concentration: 37.8 µg/mL, percentage of inhibition: 43.1) were used as positive controls. Data are expressed as mean ± standard error of mean of 3 different experiments

Figure 1 shows, respectively, the regression analysis of DPPH radical generation inhibition as a tannins concentration function in the *R. mangle* extract and its polyphenols fraction. Both, the extract and its polyphenols fraction, significantly decreased DPPH radical formation depending on the tannin concentration, with index of determination (*r*^2^) values of 0.997 and 0.998, respectively. The half inhibitory concentration (IC_50_) values of extract and its fraction were 6.7 g tannins/mL and 7.6 µg tannins/mL, respectively, demonstrating the superiority of the extract in this effect. The comparison of regression lines showed significant differences (*P* < 0.05) between them, suggesting that the extract was more effective in scavenging radical species than its fraction at tannin concentrations below the crossing point of both lines. Both samples were more effective than tested positive controls, BHT (22.0 µg/mL) and phloroglucinol (37.8 µg/mL), in trapping DPPH radicals, which showed generation inhibition values of this radical of 29.7% and 43.1%, respectively.

**Figure 2:**
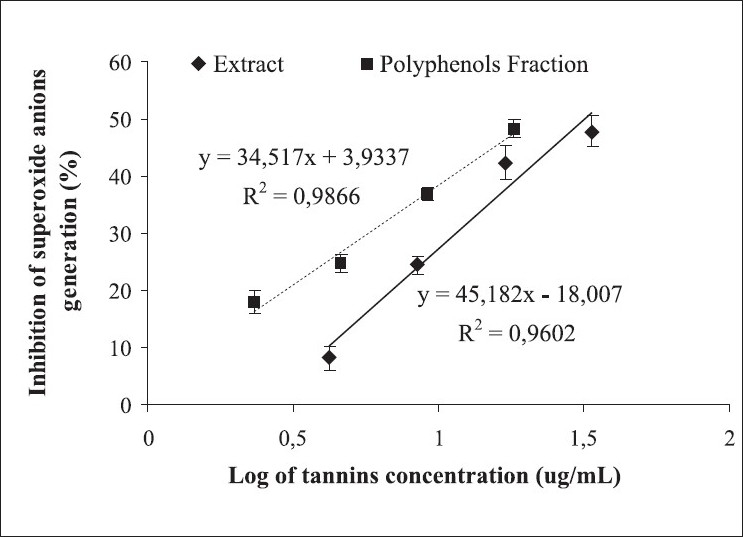
Reduction of superoxide anion formation as a tannins concentration function in the *Rhizophora mangle* L. aqueous extract and its polyphenolic fraction evaluated by xanthine– xanthine oxidase method. Data are expressed as mean ± standard error of mean of 3 different experiments.

Figure 2 shows, respectively, the regression analysis of superoxide anions generation inhibition, in the xanthine–xanthine oxidase system, as a tannins concentration function, in the *R. mangle* extract and its polyphenols fraction. It shows that generation of this free radical species decreased depending on the tannin concentration in both the samples, with *r*^2^ values of 0.960 and 0.987, respectively. The IC_50_ values of the extract and its fraction were 31.9 and 21.6 µg tannins/mL, respectively, demonstrating the superiority of the fraction in this effect. The comparison of regression lines showed significant differences (*P* < 0.05) between them, indicating that the fraction was more effective than the extract in inhibiting the production of this ROS. At the maximum concentration tested, both samples reduced approximately 50% of the production of superoxide anions.

## DISCUSSION

In the present study, the antioxidant activity of *R. mangle* extract and its polyphenols fraction was monitored, by 2 different methods, toward free radical-scavenging activity.

The spectrophotometric method of DPPH radicals scavenging has been one of the most commonly used method to estimate the antioxidant activity.[21–24] The DPPH radical is considered a stable free radical due to the delocalization of electrons available on the entire molecule, showing that the molecules do not dimerize. This electronic delocalization takes place in the radical form, which has violet color observed in the experiments and is responsible for the absorption band at 517 nm, when DPPH was prepared in ethanol solution.[25]

When the solution of DPPH radicals is mixed with *R. mangle* extract or its major fraction, it decreased the absorbance at 517 nm, indicative of the inhibition of radical species formation. The high percentages of antioxidant activity in the fraction at all tested concentrations, suggests that polyphenolic compounds contained in it contributed significantly to the effect observed in the extract. Previous papers reported this property, in different plant extracts containing polyphenolic compounds, to inactivate the DPPH radicals.[21–24,26,27]

When the DPPH radicals solution is mixed with a substance that can donate hydrogen atoms, such as polyphenolic compounds, which are good reducing agents because of the hydrogen donor properties of their phenolic hydroxyl groups,[28] obtaining the DPPH reduced form with a resulting decrease in absorbance at 517 nm. Previous studies in this type of compound revealed that certain structural features improve the efficiency in the trapping of DPPH radicals, such as an ortho-trihydric group in ring B and a galoil group in position 3 of flavanol skeleton, which increase the electronic delocalization in the molecule and it contributes to the stability of the produced radicals, resulting in an increase in the DPPH radical-scavenging capacity.[29] Representing the DPPH radical by Z^&rad;^ and the molecule donates hydrogen atoms as AH, which is responsible for inactivating free radicals formed, the primary reaction would be as follows:

Z▪ + AH = ZH + A▪

One possible explanation of higher activity of extract with respect to the fraction, in the DPPH radicals trapping ability, is the additive or synergic effect that can result from the interaction between polyphenolic constituents present in the fraction with other type of compounds present in the extract, such as essential oils, which could contribute to increase in the antioxidant activity of the extract, because there are reports in the literature about its DPPH radicals scavenging properties.[30–32]

The positive correlation observed between antioxidant activity values of the extract and its fraction, measured by the DPPH radical-scavenging capacity, and the tannins concentration in them, corresponds to the results obtained with Argentine red, pink, and white wines[33] and orange juice[34] analyzed by this method.

*R. mangle* extract and its major fraction showed an inhibitory effect on the O_2_^▪–^ generation. The high percentages of antioxidant activity of the fraction contributes to the effects observed in the extract, at all concentrations evaluated, evidencing that polyphenolic components present in the fraction are mainly responsible for the inhibitory effect on the O_2_^▪–^ production observed in the extract.

The inhibitory effect shown by the *R. mangle* extract and its polyphenolic fraction on the O_2_^▪–^ generation, in an acellular system, using the xanthine–xanthine oxidase assay, could be explained by 2 different mechanisms: the direct scavenging of this ROS or inhibition of the production of the enzyme responsible, xanthine oxidase. Several papers report that polyphenolic compounds may exert both types of effects.

Using the same experimental system of this study, a solution of β-catechin[35] and plant extracts from *Ecklonia cava* containing polyphenolic compounds[36] showed similar inhibitory effects on the O_2_^▪–^ generation. Previously, it was reported in a comparative study on the O_2_^▪–^ trapping activity of different extracts and antioxidant substances, obtaining this effect from high to low in the following order: *Ginkgo biloba* extract, pine bark extract, β-catechin, and green tea extract.[37] Another research showed O_2_^▪–^ scavenging properties of 7 pure tannins.[38] Also, a green tea extract and 7 pure tannin isolates from it showed similar effects, revealing the importance of the linkage of gallic acid to the flavanol structure for this activity, and indicating that the ortho-trihidroxy structure in ring B determines this property.[39]

Another study reported the inhibition of xanthine oxidase enzyme activity by 5 catechins present in tea, the values of inhibition constants (Ki) in M and the types of inhibition were in decreasing order: catechin (Ki = 303.95, uncompetitive), epicatechin (Ki = 20.48, mixed), epigallocatechin (Ki = 10.66, mixed), epicatechin gallate (Ki = 2.86, mixed), and epigallocatechin gallate (Ki = 0.76, competitive).[40] Another study reported that tea polyphenols and theaflavins: theaflavin, theaflavin-3-gallate, theaflavin-3,3’-digallate, and epigallocatechin-3-gallate inhibited the xanthine oxidase enzyme activity and acted like O_2_^▪–^ scavenger. The theaflavin 3,3’-digallate acted as a competitive inhibitor of the enzyme and it was the most potent inhibitor of all compounds tested.[41] Another report indicated that the polyphenol fraction of commercial cocoa also inhibited the xanthine oxidase enzyme activity in a dose-dependent manner,[42] and the same effect was observed with the procyanidins present in the grape seed.[43]

Considering the obtained results, *R. mangle* aqueous extract showed a potent antioxidant activity, achieved by the scavenging ability observed against DPPH radicals and superoxide anions. Regarding its polyphenolic composition, the antioxidant effects observed in this study are due, most probably, to the presence of polyphenolic compounds. Further investigations of individual compounds, their *in vivo* antioxidant activities, and their different antioxidant mechanisms are needed.
